# An Opportunity to Grow or a Label? Performance Appraisal Justice and Performance Appraisal Satisfaction to Increase Teachers’ Well-Being

**DOI:** 10.3389/fpsyg.2019.02361

**Published:** 2019-11-26

**Authors:** Laura Dal Corso, Alessandro De Carlo, Francesca Carluccio, Damiano Girardi, Alessandra Falco

**Affiliations:** ^1^Department of Philosophy, Sociology, Education and Applied Psychology, University of Padua, Padua, Italy; ^2^Giustino Fortunato University, Benevento, Italy; ^3^Department of Human Science (Communication, Training, Psychology), LUMSA University, Rome, Italy

**Keywords:** performance appraisal satisfaction, performance appraisal justice, teacher performance, teacher job satisfaction, teacher life satisfaction

## Abstract

Performance management is a key factor to enhance professional development and improve teaching quality. This process is successful only if teachers perceive it as fair, clear, and effective: namely, if it is satisfying. Carefully considering teachers’ attributions in the performance appraisal process is fundamental to better clarify the relations between performance management and positive individual outcomes. Therefore, this study aims to investigate the effects of perceived performance appraisal justice on teachers’ well-being, in terms of job performance, job satisfaction, and life satisfaction, hypothesizing the mediation role of performance appraisal satisfaction. Data from a sample of Italian teachers were analyzed through structural equation modeling. Results confirm the mediation role of performance appraisal satisfaction. In particular, perceived performance appraisal justice was positively associated to performance appraisal satisfaction, which, in turn, was positively associated with job performance, job satisfaction, life satisfaction. Consequently, performance appraisal satisfaction totally mediated the relations between performance appraisal justice and the outcomes considered. Findings are relevant for two reasons. First, they contribute to better understanding the performance management process in educational settings – an issue requiring further attention. Second, they contribute to highlighting the importance of performance management efficacy, which is essential not only to improve individual well-being but also to enhance teaching quality.

## Introduction

Performance appraisal is one of the most important HR management tools and its efficient implementation is one of the greatest HR professionals’ challenges, particularly in terms of validity and reliability (Gupta and Kumar, 2013; Ivaldi et al., 2015). Performance appraisal identifies the individual’s contribution to the organizational goals and establishes individual performance standards (Ikramullah et al., 2012). It can become a real job resource (Farndale, 2017). Performance appraisal is a formal assessment of what employees have performed (Snape et al., 1998). Its ultimate purpose is to allow employees to continue to improve their job performance (Selvarajan et al., 2018) and teaching innovation (Benadusi and Giancola, 2016). Performance appraisal also has specific aims, such as accountability, professional development (Delvaux et al., 2013), and organizational growth (Rubel and Kee, 2015).

Many changes have influenced European educational systems since the ‘70s, in terms of greater school autonomy and human resources management responsibility (Benadusi and Giancola, 2016). An efficient performance appraisal system has important positive effects on teachers’ professional development. Moreover, teachers are essential for better education and future workers’ growth (Ripamonti et al., 2018; Tripathi et al., 2018). Teaching is a profession with a strong sense of meaning and charged with civic responsibility: the importance of its quality is evident. For example, it allows students to emancipate from their families, internalize norms and values, and be recognized for the goals achieved (Freddano, 2016). Therefore, performance appraisal is a fundamental tool to improve not only teaching quality but also school quality. It identifies teacher’s development and training needs, while boosting various outcomes (Obasi and Ohia, 2014). For example, Robinson et al. (2008) stated that teachers’ professional development indirectly affects students’ outcomes. Teachers’ performance appraisal is a delicate process. On the one hand, it greatly influences the well-being of teachers (Borrelli et al., 2014; Benevene et al., 2018b) who can experience anxiety and pressure due to the evaluation (Benevene and Fiorilli, 2015; Girardi et al., 2015, 2018; Falco et al., 2017; Cuevas et al., 2018). On the other, many difficulties may arise, such as lack of time, lack of confidence, or lack of training (Donaldson and Mavrogordato, 2018). Since well-being is conceptualized as a self-realization, as a social integration and as positive orientation toward the task, job satisfaction, life satisfaction and job performance are often used as its indicators (Alonso et al., 2019). Teachers’ performance appraisal aims to be an objective system evaluating teachers and teaching through a supervisors’ analysis. Principals can make use of other assessments – for example by considering colleagues and students’ opinions or the teachers’ self-evaluation (Bradford and Braaten, 2018). Thus, the process will not be top-down, but accomodating and participatory, and will be free to make use of scientific methodologies (such as action-research), to establish an example of good practice for the whole school community (Freddano, 2016).

This study focuses on individual perceptions of performance appraisal: although the entire process conveys contextual factors, considering its subjective elements is of utmost importance (Kim, 2016). Reactions, perceptions, and attributions teachers make about the judgments received – in terms of fairness and satisfaction – influence their outcomes. The paper aims to bring the following contributions, based on the little evidence on performance appraisal justice (Rubel and Kee, 2015). First, its mechanism of action – e.g., mediation effects (Gupta and Kumar, 2013) – is not clear. Therefore, we aim to investigate potential mediations in the relations between performance appraisal justice and its outcomes. Second, performance management literature highlights a gap in both organizational and individual performance appraisal positive outcomes (Van De Voorde et al., 2012). Finally, we aim to clarify the relations between performance appraisal perceptions – in terms of justice and satisfaction – and some well-being dimensions, namely job satisfaction, life satisfaction, and job performance.

This study considers positive dimensions and analyzes how to enhance well-being. We draw on positive psychology framework (Seligman and Csikszentmihalyi, 2000). This movement aims to clarify and promote optimal functioning, by amplifying strengths and encouraging global well-being (Shankland and Rosset, 2017). Traditionally, psychology has focused on disease. Positive psychology is not only a research topic; it looks to goodness, both in people and in contexts (Ciarrochi et al., 2016). Emotions and positive feelings lead people to broaden and build themselves (Fredrickson, 2001), whereas their lack – or the presence of negative states – leads to failures and unhappiness. Positive organizational interventions aim to take advantage of strengths and focus on the brighter side of situations (Ghosh and Deb, 2017). Performance appraisal, if perceived as fair, allows teachers to be more flexible and efficient, and to experience the vitality promoted by positive psychology. This process is possible if the organizational interventions follow some values. Among them, Ciarrochi et al. (2016) identified self-challenge and continuous learning. We can connect the enhancement promoted by positive psychology with an important theoretical framework, the conservations of resources (COR) theory (Hobfoll, 1989, 1998). COR theory states that people are motivated to gain, maintain and nurture resources. This resource enrichment positively influences people’s well-being, whereas resource loss leads them to distress. Resources are all organizational aspects that can stimulate personal growth, learning and development (Demerouti et al., 2001), so performance appraisal justice and performance appraisal satisfaction are part of them. Therefore, based on COR theory, we wonder whether fair and satisfying performance appraisal processes can originate and enhance positive outcomes.

Among performance appraisal attributions made by workers, fairness is very important, because it substantially directs several outcomes, such as psychological contract (Barbieri et al., 2018) and efficiency (Selvarajan et al., 2018). Clear, rational, and univocal criteria allow conclusion of an efficient performance appraisal (Longenecker, 1997). Moreover, a performance appraisal perceived like a criticism – rather than an instrument for professional development – can determine teachers’ attitudes of closure (Lucisano and Corsini, 2015). Fairness regarding performance appraisal is part of organizational justice, an overarching variable formed by various sub-dimensions: distributional justice, procedural justice, and interactional justice; with the latter having been split into interpersonal and informational justice (Greenberg, 1993). According to Colquitt et al. (2001), distributional justice depends on the comparison of efforts made, rewards received, and colleagues’ rewards. Procedural justice derives from the evaluation of the processes and policies used in performance appraisal. Interpersonal justice looks to perceived dignity and respect during the feedback. Informational justice regards the information obtained on process management. Performance management quality is positively associated with positive outcomes, such as commitment and intention to remain, and is negatively associated with negative outcomes, like job stress (Su and Baird, 2017; Falco et al., 2018). In particular, a performance appraisal perceived as partial – e.g., made by biased appraisers – can originate negative consequences for the teacher. Thus, performance appraisal justice is fundamental, because it leads to various positive outcomes, such as engagement (Gupta and Kumar, 2013; Farndale, 2017), motivation to improve job performance (Selvarajan et al., 2018), pay-for-performance effectiveness (Kim, 2016), organizational commitment (Guchait and Cho, 2010), perceived organizational support (Farndale, 2017), and decreased turnover intentions (Rubel and Kee, 2015).

Satisfaction with performance appraisal is another key dimension to analyze subjective responses to performance appraisal. It is a global evaluation of the performance appraisal received, and it involves perceptions regarding one’s participation to the evaluation process, the feedback received, and its consequences on rewards distribution. Satisfaction with performance appraisal concerns various facets, such as appraiser’s trust and feedback utility (Delvaux et al., 2013). It can influence attitudes and behaviors toward the organization (Hong, 2018). When performance appraisal is congruous with individual efforts, the process is satisfying (Gözükara et al., 2017). Performance appraisal satisfaction stimulates the acknowledgment and the use of the process itself; on the contrary, a lack of performance appraisal satisfaction causes negative consequences, such as intention to quit (Guchait and Cho, 2010), work-family conflict (Ismail and Gali, 2017), and strain (Van Thielen et al., 2018). Fairness perceived on performance appraisal is fundamental to its satisfaction (Naji et al., 2015): some evidence state that performance appraisal justice is an antecedent of performance appraisal satisfaction (Lira et al., 2016; Hong, 2018). Therefore, we hypothesize that:

H1: performance appraisal justice is positively associated with performance appraisal satisfaction.

The relation between performance appraisal justice and its possible outcomes deserves an in-depth analysis. For example, the recognition of the individual contribution – also achievable through a fair performance appraisal – contributes to organizational productivity, while inadequate performance management practices lead to great productivity losses (Bloom et al., 2014). The more the performance appraisal is unfair, the less the feedback will be useful to improve job performance (Naji et al., 2015). On the contrary, fair performance appraisal improves job performance (DeNisi and Pritchard, 2006; Gruman and Sacks, 2011). Job performance is considered as a behavior completely under the control of the individual, an act of doing a job and a means to reach a set of goals within a job (Campbell, 1990). The lack of openness, legitimation and integrity, and the perception of favoritisms and biased evaluations lead to a gap between ideal job performance and real job performance (Cunha et al., 2018): employees’ resources are depleted by these negative perceptions and are not allocated to job performance anymore (Falco et al., 2013b). It is important to shed light on the mechanism by which performance appraisal justice influences outcomes, with particular reference to mediated relations (Gruman and Sacks, 2011; Gupta and Kumar, 2013; Van Thielen et al., 2018). Considering job performance, Malik and Aslam (2013) pointed out that great performance appraisal satisfaction activates a mechanism that increases job performance. Selvarajan et al. (2018) connected performance appraisal justice to job performance through the motivation to perform better. Drawing on this evidence and considering the positive relation between performance appraisal justice and job performance (Kuvaas, 2006; Aly and El-Shanawany, 2016), we would like to clarify the role of performance appraisal satisfaction in the relation between performance appraisal justice and job performance. Therefore, we assume that:

H2a: performance appraisal justice is positively associated with job performance.

H2b: performance appraisal justice is positively associated with job performance through performance appraisal satisfaction.

Job satisfaction is a key-factor for organizational success (Mufti et al., 2019). It is positively associated with goals achievement (Malik et al., 2010). This pleasant emotional state derives from individual evaluations about the job. It corresponds to the pleasantness perceived on the job and motivates people to be committed to their job activities (Karimi et al., 2011; Bélanger et al., 2015; Benevene et al., 2018a). Evidence showed the association between performance appraisal justice and job satisfaction (Salleh et al., 2013; Agyare et al., 2016; Van Thielen et al., 2018). Since job satisfaction is a global evaluation of various job features, it can be affected by the performance appraisal system. For example, in the literature abundant evidence have demonstrated the positive relation between performance appraisal satisfaction and job satisfaction (Karimi et al., 2011; Decramer et al., 2015; Van Thielen et al., 2018). Therefore, we assume that:

H3a: performance appraisal justice is positively associated with job satisfaction.

H3b: performance appraisal justice is positively associated with job satisfaction through performance appraisal satisfaction.

Looking to vocational outcomes without considering personal well-being could be a low-value choice (Van De Voorde et al., 2012). Work context and life domain are not distinct spheres: they are interdependent and could influence each other in many ways (Chummar et al., 2019). Then, we would like to take a further step: to explore the relation between performance appraisal justice and life satisfaction, which is often used as an indicator of well-being (Alonso et al., 2019). Life satisfaction is a cognitive evaluation regarding how satisfying our entire life is (Hart, 1999). Essentially, how much our life quality pleases us. Many studies stated the association between life satisfaction and job satisfaction (Aydintan and Koç, 2016; Goetz et al., 2019; Masdonati et al., 2019), that is associated with performance appraisal justice, as mentioned above. Moreover, organizational justice – the overarching dimension in which performance appraisal justice belongs – is associated with life satisfaction (Tepper, 2000; Lambert et al., 2010). Performance management can influence personal domain, as well. Dissatisfaction with performance appraisal has negative effects on personal life: for example, it increases work-life conflict (Falco et al., 2013a; Ismail and Gali, 2017; De Carlo et al., 2019). However, to the best of our knowledge, the literature has not examined the relation between performance appraisal system and life satisfaction. We suppose it can follow the performance appraisal satisfaction – job satisfaction relation. Therefore, we would like to explore the role of performance appraisal satisfaction in the relation between performance appraisal justice and life satisfaction, assuming that:

H4a: performance appraisal justice is positively associated with life satisfaction.

H4b: performance appraisal justice is positively associated with life satisfaction through performance appraisal satisfaction.

## Materials and Methods

### Participants and Procedure

Participants were directly contacted and decided to take part in the research on voluntary basis. Consequently, we gathered a convenience sample. One hundred sixty-one Italian teachers filled out the paper-and-pencil self-administered questionnaire (Table 1). The sample average age was 46.3 years (SD = 10.11) and gender distribution was 63.4% women and 36.6% men. With regards to educational levels, 89.4% of participants held a degree, 5.6% held a high school diploma, 0.6% completed only middle school, and 4.3% held a further kind. Most of the participants had been working for the same school for more than 4 years (74.5%), 22.4% had been working for a period ranging between 2 and 4 years, and 3.1% had been working for less than 1 year. The majority of the sample is full-time employed (88.2%), whereas 10.6% is part-time employed (1.2% missing). 90.7% had an open-ended contract (or apprenticeship) and 9.3% had a fixed-term contract (or replacement). All participants gave their written informed consent before the administration of the questionnaire, in accordance with the Declaration of Helsinki. The study was carried out in accordance the rules of AIP (Associazione Italiana di Psicologia – Italian Association of Psychology), according to which there was no need for previous ethics approval, since it would not deal with animals or vulnerable groups, or would involve risk for the well-being of participants, or use biomedical devices, or invasive investigation tools. Our study was conducted in accordance with the recommendations of the Ethic Committee of Psychology Research of the University of Padua, with the above-mentioned written informed consent from all participants.

**TABLE 1:** Sample characteristics.

	***M***	**SD**	**Minimum**	**Maximum**

**Age**	46.3	10.11	25	65

		**Valid %**	***N***	**Valid %**

**Gender**			**Current working time**	

Female		63.4	Full-time	88.2
Male		36.6	Part-time	10.6
Total		100.0	Missing	1.2
			Total	100.0

**Educational level**			**Type of contract**	

University		89.4	Open-ended contract or apprenticeship	90.7
High school		5.6	Fixed-term contract or replacements	9.3
Other		4.3	Total	100.0
Junior high school		0.6		
Total		100.0		

**Duration of service**				
Over 4 years		74.5		
2–4 years		22.4		
Under 1 year		3.1		
Total		100.0		

### Measures

*Performance appraisal justice* was assessed with 17 items (Gupta and Kumar, 2013) adapted from Colquitt et al. (2001). The scale measured four dimensions: distributive justice (e.g., “The outcome of performance appraisal is appropriate for the work I completed”), procedural justice (e.g., “The procedures followed during performance appraisal process are free of bias”), interpersonal justice (e.g., “My supervisor treated me with dignity during the performance appraisal meeting”), and informational justice (e.g., “My supervisor explained the procedures of the performance appraisal process thoroughly”). The 5-point response scale ranged from 1 (strongly disagree) to 5 (strongly agree). The Cronbach’s alpha for the scale is 0.95.

*Performance appraisal satisfaction* was assessed with six items (Kuvaas, 2006): global satisfaction with performance appraisal, satisfaction with the feedback received, and perceived organizational commitment to performance appraisal (e.g., “I am satisfied with the way my organization provides me with feedback,” “The feedback I receive on how I do my job is highly relevant,” “I think that my organization attempts to conduct performance appraisal the best possible way,” respectively). The items were on a 5 point-Likert scale (1 = strongly disagree; 5 = strongly agree). The Cronbach’s alpha for the scale is 0.92.

*Job performance* was assessed with two items rated on a 10-point Likert scale (from 10% to 100%): “We would kindly ask you to specify, using a percentage, how successful you were in reaching your work goals last year” and “How do you rate your job performance during the last year?” The Cronbach’s alpha for the scale is 0.85.

*Job satisfaction* was assessed by asking “How satisfied are you with your working life?” The item was taken from the Q_u_-Bo test (De Carlo et al., 2008–2011) and was on a 6 point-Likert scale (1 = very dissatisfied; 6 = very satisfied).

*Life satisfaction* was assessed by asking “How satisfied are you with your overall life?” The item was taken from the Q_u_-Bo test (De Carlo et al., 2008–2011) and was on a 6 point-Likert scale (1 = very dissatisfied; 6 = very satisfied).

### Statistical Analyses

We tested the hypotheses by means of structural equation models (SEM) with latent variables, using the Lisrel 8.80 software (Jöreksog and Sörbom, 2006). Consequently, we estimated another model, fixing all the non-significant paths to zero, to obtain a more parsimonious solution.

We assessed the model fit, starting with the chi-square test (χ^2^). A model shows a good fit to the data if χ^2^ is non-significant. Given that χ^2^ is sensitive to sample size, we considered additional fit indices. In particular, we considered the non-normed fit index (NNFI), the standardized root mean residual (SRMR), and the root mean square error of approximation (RMSEA). Values close to or greater than 0.95 for NNFI, values close to or smaller than 0.10 for SRMR, and values close to or smaller than 0.08 for RMSEA indicate an acceptable fit (Schermelleh-Engel et al., 2003).

We considered 95% asymmetric confidence intervals based on the distribution of the multiplication term, to verify the significance of the indirect effects. The purpose was to manage the non-normality derived from the *path a × path b* multiplication, as recommended by MacKinnon’s procedure (PRODCLIN; MacKinnon et al., 2007). If the confidence interval does not contain zero, the indirect effect is significant (MacKinnon et al., 2012).

Before testing the model, we carried out two procedures. First, we excluded participants with missing values; therefore, the final sample consisted of 154 participants. Second, we tested if common method variance (CMV) was a threat to the study. In fact, as we collected data through self-report measures, the risk of CMV may exist. Therefore, we controlled for the effects of a latent method factor, by using a single-common-method-factor approach in a confirmatory factor analysis (CFA) (Podsakoff et al., 2003). We added a new latent variable called “method” on which we loaded all indicators of the five theoretical factors. Consequently, we obtained a six-factor model. Then, we compared χ^2^ of the six-factor model to the five-factor model’s one. If the *p*-value associated with Δχ^2^ is significant, the effect of the latent method factor exists. To evaluate the impact of this effect, we partitioned the observed variance of the indicators into three component: variance attributable to the theoretical factors, to the method and to the causal error. In particular, Williams et al. (1989) identified the following average partitioning: 50% variance attributable to the theoretical factors, 27% variance attributable to the method, and 23% variance attributable to the causal error. If the variance attributable to the method is up to 27%, the CMV does not lead to inaccurate results.

## Results

### Descriptive Results

Table 2 presents means, standard deviations, and correlations. The latter provide initial evidence that all variables could be positively associated to each other.

**TABLE 2:** Means, SD, and correlations among study variables.

**Variable**	***M***	***SD***	**1.**	**2.**	**3.**	**4.**	**5.**
1. Performance appraisal justice	3.60	0.82	1.00				
2. Performance appraisal satisfaction	3.33	1.01	0.72^∗∗^	1.00			
3. Performance	7.94	1.09	0.40^∗∗^	0.50^∗∗^	1.00		
4. Job satisfaction	4.84	0.93	0.21^∗∗^	0.29^∗∗^	0.64^∗∗^	1.00	
5. Life satisfaction	5.05	0.94	0.17^∗∗^	0.32^∗∗^	0.36^∗∗^	0.52^∗∗^	1.00

**N* = 161, ^∗∗^*p* < 0.01.*

### Common Method Variance

The comparison between the χ^2^ of the two alternative models suggests the possible existence of a method effect (Δχ^2^ = 88.45; Δdf = 14; *p* = 0.00). Even though the variance partitioning indicated that the variance attributable to the method exists, this is limited and accounts for 17% of the total observed variance. Therefore, these results suggest that CMV is not a concern in this study.

### Model Testing

We estimated the hypothesized structural model that satisfied the acceptability criteria [χ^2^(69) = 150.83, *p* = 0.00; NNFI = 0.96; SRMR = 0.05; RMSEA = 0.08]. However, the direct relations between performance appraisal justice and the outcomes – namely job performance, job satisfaction, and life satisfaction – were non-significant (*H2a*, *H3a*, *H4a* rejected). To obtain a more parsimonious solution, we then set all these paths to zero.

**FIGURE 1:**
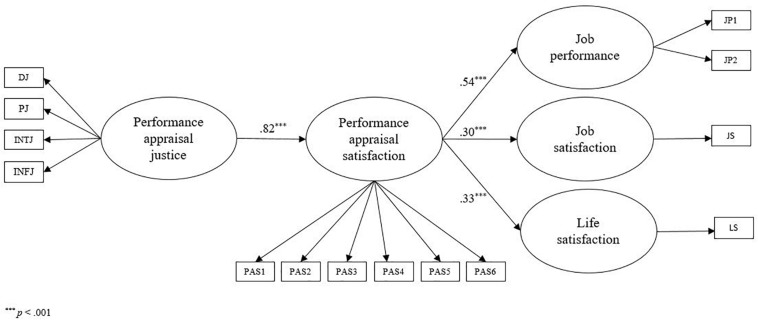
The final model.

This second model tests the total mediation of performance appraisal satisfaction. The model (Figure 1) shows an acceptable fit to the data, considering χ*^2^*(72) = 152.97 (*p* = 0.00); RMSEA = 0.08; NNFI = 0.97; and SRMR = 0.05. In the model, performance appraisal justice is positively associated with performance appraisal satisfaction (γ = 0.82, *p* < 0.001). Therefore, H1 is confirmed. Furthermore, performance appraisal satisfaction is positively associated with job performance (β = 0.54, *p* < 0.001), job satisfaction (β = 0.30, *p* = 0.001), and life satisfaction (β = 0.32, *p* < 0.001).

At this point, we verified the significance of the indirect effects. The asymmetric confidence intervals for the relationships between performance appraisal justice and the outcomes, through performance appraisal satisfaction, do not contain zero. We can conclude that performance appraisal satisfaction totally mediates the relationship between performance appraisal justice and the outcomes (*H2b*, *H3b*, *H4b* confirmed). In particular, the unconventional estimate is 0.65, 95% CI [0.41028,0.91924] for the relationship between performance appraisal justice and job performance. The unconventional estimate is 0.34, 95% CI [0.15612,0.55012] for the relationship between performance appraisal justice and job satisfaction. The unconventional estimate is 0.38, 95% CI [0.18804,0.58972] for the relationship between performance appraisal justice and life satisfaction. Consequently, we can conclude that performance appraisal satisfaction totally mediates the relationship between performance appraisal justice and the outcomes.

## Discussion

The study had the following aims. First, we intended to investigate the role of performance appraisal perceptions in a teachers’ sample – in terms of justice and satisfaction – in enhancing well-being outcomes. Second, we aimed to clarify its influence mechanisms on the outcomes, particularly mediation.

High-quality teaching allows schools to focus on students’ skills and knowledge, to educate future citizens, and promote social inclusion (Barone, 2016). Performance appraisal systems permit not only to regulate educational system, but also to create continuous innovation and improvement (Freddano, 2016). Performance appraisal is a process critical to teaching quality: if perceived as inadequate, it has negative consequences. The performance appraisal should not be a mere red-tape fulfilment: it should be carried out in depth, to actually influence school functioning (Barone, 2016). According to Cunha et al. (2018) some believe that it would be better to eliminate this appraisal system, because of its weaknesses, such as the excessive focus on the dyadic and unbalanced relation between appraiser and appraisee, and the thorny reference to meritocracy. Furthermore, performance management system is inefficient if openness is lacking and integrity is not perceived. For this reason, perceiving justice in performance appraisal is a key factor to enhance positive outcomes.

Our findings confirm the importance of positive perceptions in performance appraisal. Indeed, performance appraisal justice enhances well-being outcomes, namely job performance, job satisfaction, and life satisfaction. However, this improvement is possible only through satisfaction with performance appraisal, because our findings show that the direct relationships between performance appraisal justice and the outcomes are non-significant. Satisfaction with performance appraisal is needed, because it totally mediates the relations between performance appraisal justice and the outcomes. Finally, our study confirms that performance appraisal justice and performance appraisal satisfaction affect job performance (Gruman and Sacks, 2011; Malik and Aslam, 2013) and job satisfaction (Decramer et al., 2015; Agyare et al., 2016). Moreover, we argue that performance appraisal justice and performance appraisal satisfaction have non-vocational outcomes, namely life satisfaction.

Therefore, performance appraisal appropriateness, unbiased procedures, dignity during performance appraisal meetings, and adequate explanation of the procedures used – namely, performance appraisal justice (Gupta and Kumar, 2013) – is not sufficient to increase the positive outcomes. Performance appraisal satisfaction is fundamental to reach this goal. For this reason, performance appraisal has to be perceived as a global, positive process: its perceived justice is important together with its other facets, such as the relevance of the feedback received, the right recognition of the individual performance, and the organizational engagement in providing a constructive feedback – aspects constituting performance appraisal satisfaction (Kuvaas, 2006). Thus, according to our results, all the aspects of performance appraisal satisfaction convey performance appraisal justice.

The study has some limitations. First, its cross-sectional nature does not allow us to determine the direction and the causality of the relations. Even if strong reasons support our results, future research may choose to conduct longitudinal studies. Since we used only one data gathering method, future research could take into account different kinds of evaluations (Falco et al., 2013c; Girardi et al., 2019); for example, they can consider students’ and colleagues’ perspectives. Drawing on the importance of subjectivity, future research could consider the moderation of some individual characteristics, such as optimism or self-efficacy, or some personality traits (e.g., perfectionism, need for cognitive closure; Falco et al., 2014; Bélanger et al., 2016). Moreover, since contextual variables could explain the results trend, future research could take into account the school level as an independent variable. Finally, in the future it is worth examining how to promote performance appraisal justice, to make it an integral part of the educational system. It is also essential to reflect on the real purposes of the appraisal system (Maccarini, 2016), that is to adjust to match those of the educational system, and the overall quality-equity binomial (Benadusi and Giancola, 2016).

## Conclusion

The study underlines the relationships between performance appraisal justice and some positive individual outcomes (e.g., performance, job satisfaction, life satisfaction). These relationships are totally mediated by performance appraisal satisfaction. The study gives the following contributions. First, in examining the performance appraisal system, it considers its perceptions and its subjective facets, rather than the organizational ones. In doing so, it sheds light on the relationships between these perceptions and the positive outcomes considered. Second, it clarifies the mechanisms of action of performance appraisal justice, which was not clear (Rubel and Kee, 2015). Third, it identifies a possible antecedent of performance appraisal satisfaction (Ismail and Gali, 2017). Fourth, it represents – to the best of our knowledge – a first step in the study of the effects of the performance appraisal system perception on life satisfaction.

## Practical Implications

Based on our results, justice and satisfaction are basic facets of the performance appraisal system. Therefore, planning training activities would be advisable to strengthen the skills that principals use during these performance appraisal meetings. The interventions could concern communication, soft, and positive, managerial skills, and psychological counseling (Dal Corso et al., 2013; Scaratti and Ivaldi, 2015; De Carlo et al., 2016; Farnese et al., 2017). These competences could help teachers to perceive performance appraisal as a constructive, encouraging process.

## Data Availability Statement

The datasets generated for this study are available on request to the corresponding author.

## Ethics Statement

Ethical review and approval was not required for the study on human participants in accordance with the local legislation and institutional requirements. The patients/participants provided their written informed consent to participate in this study.

## Author Contributions

LD developed the research project, with the contribution of AD, AF, and DG. LD reviewed the literature, with the contribution of AD and AF. FC prepared the data set. DG and FC carried out the data analysis.

## Conflict of Interest

The authors declare that the research was conducted in the absence of any commercial or financial relationships that could be construed as a potential conflict of interest. The handling Editor declared a shared affiliation, though no other collaboration, with one of the authors FC at the time of review.
